# Reference gene selection for the shell gland of laying hens in response to time-points of eggshell formation and nicarbazin

**DOI:** 10.1371/journal.pone.0180432

**Published:** 2017-07-03

**Authors:** Sami Samiullah, Juliet Roberts, Shu-Biao Wu

**Affiliations:** Animal Science, School of Environmental and Rural Science, University of New England, Armidale, New South Wales, Australia; Northwestern University Feinberg School of Medicine, UNITED STATES

## Abstract

Ten reference genes were investigated for normalization of gene expression data in the shell gland of laying hens. Analyses performed with geNorm revealed that hypoxanthine phosphoribosyltransferase 1 (*HPRT1*) and hydroxymethylbilane synthase (*HMBS*) were the two most stable reference genes in response to post-oviposition time alone (POT) or with nicarbazin treatment (POT+N) of laying hens. NormFinder analyses showed that the two most stable reference genes in response to POT and POT+N were 18S ribosomal RNA (*18S rRNA*), ribosomal protein L4 (*RPL4*) and *HMBS*, *RPL4*, respectively. BestKeeper analyses showed that *18S rRNA*, *RPL4* and *HPRT1*, *HMBS* were the two most stable reference genes for POT, and POT+N, respectively. Of the ten reference genes, all except *B2M* showed geNorm M <0.5, suggesting that they were stably expressed in the shell gland tissue. Consensus from these three programs suggested *HPRT1* and *HMBS* could be used as the two most stable reference genes in the present study. Expression analyses of four candidate target genes with the two most and the two least stable genes showed that a combination of stable reference genes leads to more discriminable quantification of expression levels of target genes, while the least stable genes failed to do so. Therefore, *HMBS* and *HPRT1* are recommended as the two most stable reference genes for the normalization of gene expression data at different stages of eggshell formation in brown-egg laying hens. Available statistical programs for reference gene ranking should include more robust analysis capability to analyse the gene expression data generated from factorial design experiments.

## Introduction

The chicken reproductive tract is divided into five histologically distinct parts, the ovary, infundibulum, magnum, isthmus, and shell gland (uterus). The shell gland is an expanded pouch-like part of the oviduct where an egg remains for approximately 18–20 hours, during which shell formation takes place [1]. The next ovulation occurs 0.5 hour after the preceding oviposition [2]. Calcification of the eggshell is associated with stimuli initiated by ovulation or by neuroendocrine factors that control and coordinate both ovulation and calcium secretion [3]. The eggshell is a highly ordered bio-ceramic (about 5 g calcium) of fused calcite crystalline cones, formed on a protein skeleton with distinct layers and regular pores [4]. Like other epithelial cells, the shell gland epithelium provides antimicrobial protection for both hen and egg; thus, it is a rich source of anti-microbial proteins [5].

Approximately 437 peptides and ion transporters have been identified as being involved in the formation of three distinct layers of the eggshell [6, 7]. However, the mechanisms of egg and eggshell biogenesis in relation to the origin and flow of the precursors at various stages of egg formation are not fully understood. The biosynthetic pathway of protoporphyrin IX (PP IX) is important in the formation of the eggshell as it contributes to the shell colour. PP IX is an immediate precursor of heme and a major component of brown eggshell pigment. To date, there is little information about the origin of its precursors and how PP IX is inhibited from converting into heme through the enzymatic activity of ferrochelatase in the shell gland of laying hens. Furthermore, it is not clear how many genes are involved in the PP IX synthesis, ultimate transportation across cell membranes and subsequent deposition into distinct eggshell layers. In the shell gland, hundreds of genes that are differentially expressed between juvenile and mature laying hens have been identified [8]. It is assumed that some genes express differentially in relation to the formation of distinct layers of the eggshell. Furthermore, the expression of genes associated with epithelial differentiation and tissue remodelling may vary with different levels of estrogen secretion in the presence or absence of an egg [9].

Nicarbazin is one of the various factors that causes lower production and/or deposition of PP IX into eggshell, when fed at recommended dosages (50–125 mg/kg of feed) to brown-egg laying hens [10, 11]. It is a chemically produced drug composed of a complex equimolar amount of 4,40-dinitrocarbanilide (DNC) and 2-hydroxy-4,6-dimethylpyrimidine (HDP) and is registered for use in poultry fattening and for treating coccidiosis [10, 12]. It is used either in pure or combined forms as a feed additive in poultry production [13]. Its effects on eggshell colour, which are dosage and time dependent, are reversible [14]. The pharmacodynamics of nicarbazin in the shell gland are not yet known, and the way it acts at the molecular level to alter the synthesis and/or deposition of PP IX into eggshells at different stages of shell formation needs to be further investigated. Therefore, we anticipate that the responses of the genes involved in the PP IX synthesis pathway to nicarbazin will shed some light on its actions in the alteration of eggshell colour deposition. Nicarbazin was used as a model as residues of this drug in feed are still a problem of loss in shell colour particularly in Australia. In addition, investigation of the molecular basis of nicarbazin effects on shell colour may shed light on the mechanisms of shell colour loss in commercial laying flocks from other causes.

The transcriptional profiling in the shell gland is different from other parts of the oviduct, such as the magnum and isthmus [7]. Thus, data on differential gene expression in the shell gland are needed and, therefore, selection of reference genes for gene expression analysis as a normalization approach is paramount. To the best of our knowledge, no studies have been performed to identify suitable reference genes for the normalization of quantitative PCR (qPCR) gene expression data in the shell gland of chickens. Traditionally, the most commonly used housekeeping genes, such as *ACTB*, *TUBB*, and *GAPDH* have been selected as generic reference genes. However, ample evidence has shown that the expression of these genes may not be constant across a range of experimental conditions and tissues under investigation [15–20]. Thus, it is now recommended to use these genes only as reference genes for normalization when prior analysis of their expression stability has been carried out [21], to ensure that cellular expression level of the reference genes is virtually identical under different conditions in the study. It is also recommended that more than one reference gene be employed to achieve more robust, accurate and reliable normalization of gene expression data [22].

Of the available statistical software, three distinct tools have been frequently reported in the literature for ranking the overall expression stabilities of the reference genes as normalizers for gene expression studies. The geNorm module in qbase+ software version 3.0 (Biogazelle, Belgium) calculates the gene expression stability (geNorm M) as the arithmetic mean of the pairwise variation (geNorm V) between all tested genes [22, 23]. The geNorm V for any given two genes is the standard deviation calculated from the log_2_ transformed relative quantities between those two genes [22]. Before analysis, qbase+ pre-processes the data for efficiency correction, inter-run calibration, bad replicates removal and conversion to relative quantities [23]. The relative quantities are then converted to either linear or log transformed scale. The current geNorm tool does limit the minimum number of genes to 8, unlike to its previous Excel based version. qbase+ allows easy exchange of data between users, and exports tabulated data for further statistical analysis using dedicated software. The most stably expressed gene produces the lowest geNorm M value. The most stable reference gene is determined by step-wise exclusion of the least stable genes [22]. geNorm eliminates the genes sequentially and thus a differentially expressed gene does not affect the ultimate outcomes from the analysis. Therefore, geNorm is usually less sensitive to differentially expressed genes initially included in the assay. Good reference genes have an M < 0.5 and CV (Coefficient of variance) <0.2, while M values up to 1 are acceptable for more difficult samples [23]. The cut-off value for geNorm V is 0.15. geNorm does not consider treatment groups and all samples are treated as being from a single population. NormFinder (GenEx version 6.0.1) calculates the standard deviation (SD) of the genes relative to the mean expression of all the genes in the panel [24]. It calculates a global average expression of all the genes in all the samples, to which the individual genes are compared. Based on this comparison, SD for each reference gene is estimated. Furthermore, if the samples are from different treatment groups, NormFinder separates the variation into an intragroup and an intergroup contribution [24, 25]. Hence, a low stability value reflects low inter- and intra-group variation. Similar to qbase+, GenEx does have an option to highlight bad replicates during data analysis and thus bad replicates can be excluded from the analysis easily. In the GenEx version 6.0.1, the data pre-processing is very similar to that explained in the qbase+ section. In addition, the data in GenEx, can be also converted to logarithmic scale, such as log_2_, log_10_, 1n and log(X+1). An Excel based BestKeeper (Version 1) software is used to determine the best stable reference genes based on Pearson correlation coefficient (r), coefficient of variance (CV) and standard deviation (SD). Only genes with a high r and low SD values are combined into BestKeeper index (BKI) value using the geometric mean of their Cq values [26]. The BKI is calculated from the geometric mean of the candidates Cq values for each specific sample [26]. The most stable reference genes are the ones with the lowest SD values and highest coefficients of correlation with the BKI [26]. BestKeeper also uses a statistical algorithm wherein the Pearson correlation coefficient for each candidate reference gene pair is calculated along with the probability of correlation significance of the pair [26]. Overall, a generalized opinion from the literature and scientific forums can be summarised: geNorm, NormFinder and BestKeeper basically provide similar outcomes for the overall stability of candidate reference genes.

In the present study, we aimed to select reference genes from ten housekeeping genes to be used for the analysis of gene expression levels at different stages of eggshell formation in the shell gland and in response to nicarbazin feeding of the laying hens. The most stable genes were selected on the basis of the stability of the genes across the three software. Furthermore, four candidate target genes encoding either enzymes or peptide transporter were chosen to compare the outcomes from the data by the two most and the two least stable reference genes. The solute carrier family 25, member 38 (*SLC25A38*), located on mitochondrial membrane, transports glycine into mitochondria for the synthesis of aminolevulinic acid, the first step in the synthesis of PP IX [27]. The delta-aminolevulinate synthase 1 (*ALAS1*) gene encodes a rate limiting non-erythroid enzyme that catalyses the reaction of succinyl co-enzyme A with glycine to form delta-aminolevulinic acid within the mitochondrial matrix [28]. Coproporphyrinogen oxidase (*CPOX*) gene encodes an enzyme in the PP IX biosynthetic pathway that converts coproporphyrinogen III into protoporhyrinogen III [29]. Ferrochelatase (*FECH*) gene encodes *FECH* enzyme, which converts PP IX into heme [30, 31]. The outcome of the study provides a set of reference genes that are expressed in a relatively constant level for all the birds sampled at different time-points of eggshell formation and in response to treatment with nicarbazin.

## Materials and methods

The experimental setup was approved by the University of New England, Animal Ethics Approval Committee under Authority No. AEC15-022. The protocol was carried out in accordance with the guidelines specified in the Australian Code for the Care and Use of Animals for Scientific Purposes 8^th^ edition 2013.

### Selection of reference genes and primer design

In the current study, ten reference genes were selected from the literature published for chickens and other animals (Table 1). The primers were either sourced from previously published studies in chickens or designed using NCBI primer tool (Table 2). The primer quality was checked in “Beacon Designer” software (http://www.premierbiosoft.com/qOligo/Oligo.jsp?PID=1) for the levels of secondary structures such as primer dimer, sequence repeats and palindrome. To check the sequence specificity, primers were blasted against the NCBI database using BLASTN, Ensemble Chicken Galgal4 and UCSC’s Chicken (*Gallus gallus*) Genome Browser Gateway. Prior to qPCR analysis, primer efficiency and specificity for each primer pair were examined with target RNA samples in 10-time serial dilutions. Only primer pairs with specific amplifications and high efficiency were used in the optimisation.

**Table 1:** Functional annotations of the reference genes used in the current study.

Gene symbol	Description	Cellular localization	Biological function
***18S rRNA***	Nuclear ribosomal RNA small subunit	cytoplasm, nucleus	biogenesis and export of the 40S ribosomal subunit
***ALB***	Albumin	extracellular	stabilizing extracellular fluid volume
***ACTB***	β-actin	cytoplasm, membranes	cytoskeletal structural protein, nucleotide, and ATP binding
***B2M***	Beta 2-microglobulin	Golgi membrane, plasma membrane, early endosome membrane, extracellular region	cytoskeletal protein, immune response, protein binding
***CA2***	Carbonic anhydrase 2	cytoplasm, cell membrane	catalyses reversible hydration of carbon dioxide
***CST3***	Cystatin C	extracellular	as an inhibitor of cysteine proteinases
***GAPDH***	Glyceraldehyde 3-phosphate dehydrogenase	plasma membrane	glycolytic enzyme, oxidoreductase in glycolysis and gluconeogenesis
***HMBS***	Hydroxymethylbilane synthase	cytoplasm	heme synthesis, porphyrin metabolism, transferase activity
***HPRT1***	Hypoxanthine phosphoribosyltransferase 1	cytoplasm	purine synthesis in salvage pathway
***RPL4***	Ribosomal protein L4	cytoplasm	component of the 60S subunit and encodes a ribosomal protein

**Table 2:** Forward (F) and reverse (R) primer sequences of the selected candidate reference and target genes.

Gene	Primer sequence (5′-3′)	Amplicon size (bp)	Ta (°C)	Accession No.	Reference
***18S rRNA***	F: TGTGCCGCTAGAGGTGAAATT	63	60	AF173612.1	[32]
	R: TGGCAAATGCTTTCGCTTT				
***ALB***	F: CCTGGACACCAAGGAAAT	197	60	NM_205261.2	[33]
	R: TGTGGACGCCGATAGAAT				
***ACTB***	F: CTGTGCCCATCTATGAAGGCTA	139	60	NM_205518.1	[33]
	R: ATTTCTCTCTCGGCTGTGGTG				
***B2M***	F: CGTCCTCAACTGCTTCGTG	194	63	NM_001001750.1	[33]
	R: TCTCGTGCTCCACCTTGC				
***CA2***	F: TCAAACCAAGGGGAAACAAGC	99	63	NM_205317.1	this study
	R: GTAGTCAGGGAGCCAGGGTA				
***CST3***	F: ATGAGAACGACGAGGGCTTG	130	63	NM_205500.2	this study
	R: ATTCCAGACACGAGCTGCC				
***GAPDH***	F: GAGGGTAGTGAAGGCTGCTG	113	63	NM_204305.1	[34]
	R: CATCAAAGGTGGAGGAATGG				
***HMBS***	F: GGCTGGGAGAATCGCATAGG	131	60	XM_417846.2	[35]
	R: TCCTGCAGGGCAGATACCAT				
***HPRT1***	F: ACTGGCTGCTTCTTGTG	245	63	NM_204848.1	[33]
	R: GGTTGGGTTGTGCTGTT				
***RPL4***	F: TTATGCCATCTGTTCTGCC	235	60	NM_001007479.1	[32]
	R: GCGATTCCTCATCTTACCCT				
SLC25A38*	F: AGACACGGTATGAGAGTGGA	139	63	XM_418818.3	this study
	R: ATCCCAGAGAAAGGTGCGTC				
CPOX*	F: GAGAGGACGGTATGTGGAGT	187	60	XM_004938236.1	[36]
	R: TTTGGGATTGCGGAGAAC				
ALAS1*	F: GGTGGACAGGAAAGGTAAAGA	197	60	NM_001018012.1	[36]
	R: ACTGGTCATACTGGAAGGTG				
FECH*	F: TGCTTTGCCGATCACAT	112	60	U68033.1	[36]
	R: CACGGTTCACCACAGACAT				

*Genes used as candidate target genes for validation of reference genes at three different time-points of eggshell formation

### Laying hens and tissue sampling

#### Effect of time-points on stability of reference gene expression (Experiment 1)

Based on the intensity of brown eggshell colour and uniformity in egg weight, 20 hens out of a flock of 63 Hy-Line Brown laying hens were selected. The laying production of the selected hens was 100%. The feed offered was premium top layer mash (Barastock, Australia). At the time of the experiment, hens were 36–37 weeks old. From the selected 20 hens, eggshell colour (L*) and egg weight (g) were measured using a Spectrophotometer (Konica CM-2600d Ramsey, NJ, USA) [37] and analytical weighing balance Quintix513-1S (Sartorius Lab Instruments GmbH & Co. KG Goettingen, Germany), respectively. The hens were divided into four groups in such a way that the average L* values and egg weight were not significantly different among the selected groups (Table 3). Individual hen oviposition time was monitored using a video camera at the time of sampling. Four groups of hens were sampled based on time-points (post-oviposition time 2, 5, 15 and 23.5 hrs). The hens were euthanized by CO_2_ and the shell gland tissue was excised within 2 minutes of the euthanization. Approximately 500 mg tissue was taken from the centre of the shell gland after opening the shell gland from the anterior-ventral position and transferred directly to RNALater (Sigma Aldrich, Australia). The samples were stored at -20°C until further processing for total RNA extraction.

**Table 3:** Eggshell colour (L*) and egg weight of the hens selected for reference gene study optimisation at different time-points. The eggs were collected and analysed before dividing the experimental hens into various groups. On the basis of eggshell variables, hens were divided into groups in such a way that the variables were not significantly different among groups.

Variable	Time-point (hr)				P value
	2 (5 hens)	5 (5 hens)	15 (5 hens)	23.5 (5 hens)	
L* value	56.24±0.99	54.38±0.98	54.19±0.89	54.36±0.28	0.2982
Egg weight (g)	62.81±3.51	63.95±2.31	62.96±1.66	65.65±1.77	0.8326

Values are mean ±S.E.

#### Effect of time-points and nicarbazin on stability of reference gene expression (Experiment 2)

A total of 30 hens having 100% laying efficiency were selected based on the intensity of eggshell colour (L*) and egg weight from the remaining flock of 43 Hy-Line Brown laying hens (Table 4). Rearing conditions were the same as described previously. At the time of the experiment, hens were 42–45 weeks old and were divided into groups with a 2×3 factorial design (Table 4). The hens were divided into groups in such a way that the average L* values and egg weight (g) were not significantly different among groups. Out of 30 hens, 15 hens were fed nicarbazin @100mg/kg of commercial layer diet while the control hens were fed only commercial layer diet. Nicarbazin was fed to each group at a time in order to allow time for the processing of the treated hens without intoxicating them. The eggshell colour (L*) and egg weight were recorded for all the hens from prior to treatment until the tissue collection. Procedures for tissue collection and handling were the same as mentioned previously.

**Table 4:** Eggshell colour (L*) and egg weight of the hens selected for reference gene optimisation with nicarbazin treatment at different time-points. The eggs were collected and analysed before dividing the experimental hens into various groups. On the basis of eggshell variables, hens were divided into groups in such a way that the variables were not significantly different among groups.

Variable	Group							
	Control (15 hens)				Nicarbazin (15 hens)			
	Time-point (hr)			P value	Time-point (hr)			P value
	5 (5hens)	15 (5hens)	23.5 (5hens)		5 (5hens)	15 (5hens)	23.5 (5hens)	
L* value	58.69±0.59	59.54±0.89	59.39±0.63	0.6827	58.46±0.67	58.89±0.52	59.06±0.51	0.7548
**Egg weight (g)**	63.73±1.77	63.29±1.58	63.87±1.58	0.6969	58.74±1.69	60.93±1.41	62.65±1.19	0.2019

Values are mean ±S.E.

### Total RNA extraction and purification

Total RNA was extracted using TRIsure (Bioline, Australia), according to the manufacturer's instructions. Briefly, an approximately 50 mg of tissue (wet weight) was homogenized in 1 mL of TRIsure using an IKA T10 basic Homogenizer (Wilmington, NC, USA). After the RNA pellet was washed with 75% ethanol and subsequently air-dried for 10–15 minutes, 50 μL of UltraPure^™^ DEPC-Treated water (Ambion, USA) was used to dissolve RNA pellets. The total RNA was further purified using RNeasy Mini Kit (Qiagen, GmbH, Hilden, Germany) as per the manufacturer’s instructions. The elution of RNA from the spin column with 50 μL of RNase-free water was repeated twice and the eluted RNA solutions were mixed thoroughly. The purified RNA was analysed in a NANODROP-8000 spectrophotometer (ThermoFisher Scientific, Wilmington, DE, USA) to measure its quantity and purity. RNA integrity was examined using 1.5% agarose gel electrophoresis in 1× UltraPure^™^ TAE Buffer (ThermoFisher Scientific, Australia) solution. The 0.1–2 Kb RNA Ladder (Ambion^™^, Australia) was used as a marker. A 1:1000 Lonza GelStar^™^ Nucleic Acid Gel Stain in Orange G loading dye was used to stain RNA and the gel was viewed and photographed using a camera. Only RNA showing two distinct 28S and 18S bands without smear was regarded integrate and used in downstream qPCR assay.

### Quantitative PCR

qPCR was performed with the SensiFAST SYBR^®^ Lo-ROX One-Step RT-PCR Kit (Bioline, Australia). Master mix was prepared as per the manufacturer’s protocol and 4 μL of RNA template from 1:100 dilutions with the exception of *18S rRNA* (that was in 10^−4^ dilutions) was added to the reaction wells using Corbett CAS1200 robotics (Corbett Life Science, Sydney, Australia). The reaction was run in triplicates of 20 μL in a Rotor- Gene Disc 100 (Qiagen, Sydney, Australia) with a Rotor-Gene 6000 thermocycler (Corbett Research, Sydney, Australia). No template control (NTC) and no reverse transcriptase (-RT) control were also included to detect possible contamination. Thermocycling conditions for a 2-step PCR were: reverse transcription at 45°C for 10 minutes, first denaturation at 95°C for 2 minutes, then 40 cycles of denaturation at 95°C for 5s and annealing at 60°C or 63°C for 20s. The fluorescent data were acquired at the end of each annealing step during PCR cycles. A melting step was conducted to assess the specificity of PCR amplification. The PCR products were examined on Agilent 2100 Bioanalyzer (Agilent Technologies, Waldbronn, Germany) gel using DNA 1000 Kit as per the manufacturer’s instructions to estimate the size of the amplicons for specificity.

PCR amplification efficiencies and correlation coefficients (R^2^) were determined with the amplifications of a series of six 10-fold dilutions. The qPCR data of the genes were processed further when the PCR amplification efficiency was in a range of 90 to 105%, and linear correlation coefficient R^2^ > 0.980 were considered of high standard [38].

### Statistical analysis

The eggshell colour (L*) and egg weight data were analysed by Statview software (SAS Institute Inc., Version 5.0.1.0). A two-way analysis of variance (ANOVA) was conducted taking time-point and nicarbazin treatment as independent and L* and egg weight as dependent variables. Level of significance was indicated by probability of less than 5%. The Fishers LSD test was used to differentiate levels of significance between mean values.

To determine the expression stability of 10 different reference genes, the geNorm module in qbase+ software version 3.0 (Biogazelle, Belgium) was used to calculate the gene expression stability measure (geNorm M) [22, 23]. The input data for qbase+ were generated using the relative quantities based on comparative quantification cycle (Cq). To be consistent, the Cq values for *18S rRNA*, which were from 10^−4^ dilutions, were adjusted according to 1:100 dilutions. Any triplicate reaction with difference more than 0.5 cycle was excluded from the analysis. To select the most stable genes, geNorm re-calculates the M stability measures after removing the least stable genes and repeats the process until the one most stable gene remains [22, 23]. To test the minimum number of reference genes, geNorm calculates a pairwise variation (geNorm V) based on V_n/n+1_ and a higher value indicates a significant effect of additional gene on data normalization. Normally, the benefit of using an extra (n+1)th reference gene is limited as soon as the V_n/n+1_ value drops below the 0.15 threshold. Due to its sequential elimination of less stable genes so as to produce less bias on the output of analysis, this program was used as the primary base for the selection of the reference genes.

In addition, another two programs, i.e., NormFinder [24, 25] and BestKeeper [26], were used to analyse the stability of gene expression as complementary measures to safeguard the output generated from geNorm. The raw Cq values were exported and analysed in NormFinder for reference gene expression stabilities. An Excel based BestKeeper (Version 1) software was also used to determine the best stable reference genes based on Pearson correlation coefficient (r), coefficient of variance (CV) and standard deviation (SD). The most stable genes were selected on the basis of the stability of the genes across the three software.

Data for the candidate target gene expression using the two most stable and the two least stable reference genes were analysed in qbase+ by scaling the average relative quantities across all unknown samples per target gene [23, 39]. Effect of time-points on the relative expression levels of the candidate target genes was analysed using one-way ANOVA. Tukey-Kramer method was used to correct the p-value results (corrected p value < 0.05) for the pairwise group comparisons in the ANOVA test [23].

## Results

### Primers specificity and efficiency

**Fig 1:**
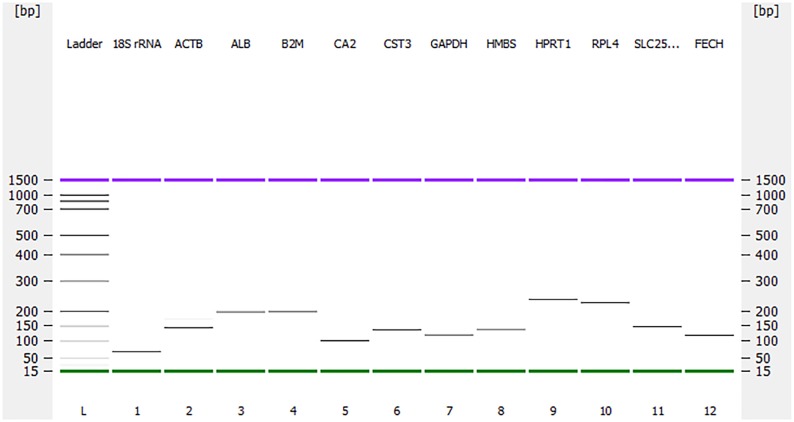
Amplification of the gene fragments from the eggshell gland tissue of laying hens to assess the specificities of the primers used in the current study. L, DNA ladder; 1. *18S rRNA* (63 bp); 2. *ACTB* (139 bp); 3. *ALB* (197 bp); 4. *B2M* (194 bp); 5. *CA2* (99 bp); 6. *CST3* (130 bp); 7. *GAPDH* (113 bp); 8. *HMBS* (131 bp); 9. *HPRT1* (245 bp); 10. *RPL4* (235); 11. *SLC25A38* (139 bp); 12. *FECH* (112 bp). All the amplified products were in accordance to the expected sizes. The amplified products were run on Agilent 2100 Bioanalyzer using Agilent DNA 1000 Kit as per the manufacturer’s instructions. The upper (purple) and lower (green) markers act as internal standards and are used to align the ladder analysis with the individual DNA sample analysis. The standard curve (plotting migration time against DNA amplicon size), in conjunction with the markers, is then used to calculate DNA fragment sizes for each well from the migration times measured (for more detail see Agilent 2100 Bioanalyzer Users Guide for Molecular Assays).

**Fig 2:**
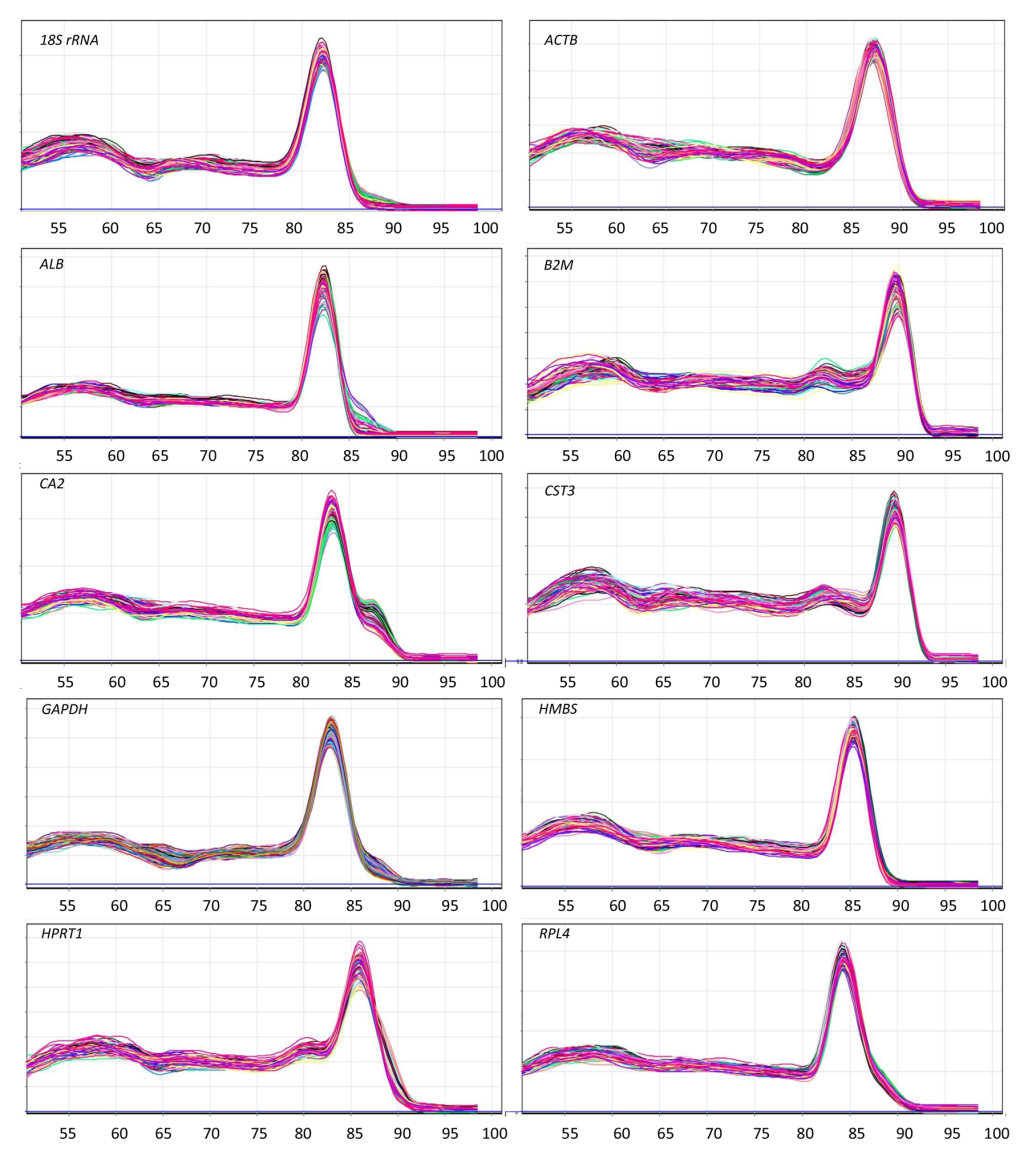
Melting curves of the amplicons from 10 candidate reference genes showing that the amplifications were specific and no primer dimers were present. All of the amplicons showed a single peak in melting curve analysis. After qPCR cycles, a melting phase at a ramp from 50°C to 99°C at 1°C increment was conducted to assess the specificity of PCR amplification.

**Fig 3:**
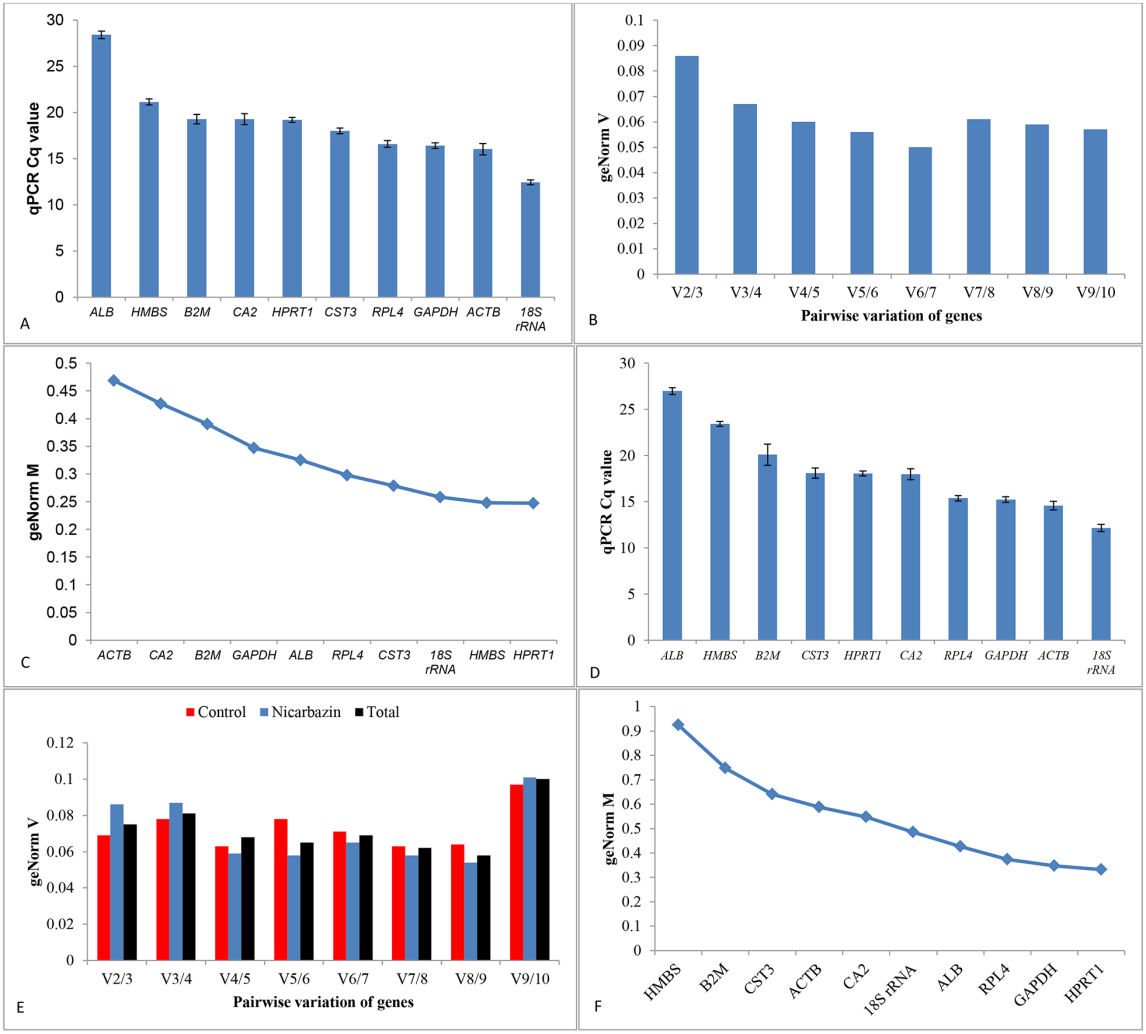
Effect of time-points and combination of time-points and nicarbazin treatment on the expression stability of reference genes in the shell gland of laying hens. A). Mean Cq values of 10 reference genes affected by time-points (Experiment 1). Bars represent standard deviation. B). Pairwise variation (geNorm V) of the optimal number of reference genes affected by time-points (Experiment 1). Pairwise variation (V_n/n+1_) was analysed between the normalization factors NF_n_ and NF_n+1_ to determine the optimal number of reference genes. In the geNorm V graph, each bar represents change in normalization accuracy by stepwise inclusion of most to least stable gene. C). Average expression stability (geNorm M) values of reference genes excluding time-point 2 (post-oviposition time, 2 hours; Experiment 1). Most stable genes have lower M value. Time-point 2 was excluded to compare it with the control group of the time-points and nicarbazin treatment experiment (Experiment 2). D). Mean Cq values of 10 reference genes affected by time-points and nicarbazin treatment (control and nicarbazin groups combined). Bars represent standard deviation. E). Pairwise variation (geNorm V) of the optimal number of reference genes (control, nicarbazin treated and both the groups combined). F). Average expression stability (geNorm M) values of reference genes (time-points (Experiment 1) and control hens from the time-points and nicarbazin treated groups (Experiment 2)). Most stable genes have lower M value.

All of the primer pairs were specific in amplifications by showing a single band on the Agilent 2100 Bioanalyzer gel (Fig 1). The melting curve analyses of all primer pairs are depicted in Fig 2. The amplification efficiency of all ten candidate reference genes was between 93% and 101%. The amplification efficiencies were 100% for *18S rRNA*, 98% for *ACTB*, 101% for *ALB*, 93% for *B2M*, 94% for *CA2*, 100% for each of *CST3* and *GAPDH*, 94% for *HMBS*, 98% for *HPRT1* and 94% for *RPL4*. The overall expression pattern (Cq values) for these ten reference genes is shown in Fig 3A. Most of the reference genes were highly expressed, with average Cq values between 12 and 22 cycles, except *ALB*, which showed average Cq values around 28 cycles (Fig 3A). The expression pattern of all ten reference genes was calculated in the combined dataset of four different time-points (2, 5, 15, 23.5 hr, post-oviposition times).

### Effect of time-points on stability of reference gene expression (Experiment 1)

Based on the expression stability (geNorm M), *HPRT1* and *HMBS* were the two most stable genes. The average expression stabilities (geNorm M) of the ten reference genes were within the acceptable range (<0.50) that varied from 0.252 (*HPRT1*) to 0.482 (*ACTB*) (Table 5). The pairwise variation (geNorm V) also chose *HPRT1* and *HMBS* as the best set of genes to be used for expression data analysis (Fig 3B). The geNorm V value of *HPRT1* and *HMBS*, which was 0.086, indicated that stepwise inclusion of the next most stable reference gene (*18S rRNA*) is not necessary for data normalization. In line with geNorm M results, all ten reference genes showed geNorm V <0.15 (a default cut-off value). The results of NormFinder and BestKeeper analyses were slightly different from those of the geNorm. NormFinder ranked *18S rRNA* and *RPL4* as the two most stable reference genes, while the two most stable reference genes in BestKeeper were *18S rRNA* and *HPRT1* (Table 5). Nevertheless, all the genes analysed by NormFinder and BestKeeper had SD < 1.0, showing that these genes were overall stably expressed in the tissue under investigation. The two least stable reference genes across all the three statistical tools were *CA2* and *ACTB* (Table 5). The expression data were also analysed in geNorm excluding post-oviposition time 2 hours but this had no significant effect on the ranking of genes (Fig 3C).

**Table 5:** Stability values of reference genes affected by four different time-points performed in Experiment 1. Means of relative expression levels of genes at four different time-points (post-oviposition times 2, 5, 15, 23.5 hrs) were used to calculate the expression stability of genes across the three statistical software.

Rank	geNorm		NormFinder		BestKeeper	
	Gene	M value	Gene	SD	Gene	SD
1	*HPRT1*	0.252	*18S rRNA*	0.065	*18S rRNA*	0.190
2	*HMBS*	0.254	*RPL4*	0.178	*HPRT1*	0.220
3	*18S rRNA*	0.264	*CST3*	0.182	*HMBS*	0.230
4	*CST3*	0.284	*HPRT1*	0.261	*CST3*	0.240
5	*RPL4*	0.306	*HMBS*	0.288	*GAPDH*	0.260
6	*ALB*	0.331	*ALB*	0.331	*RPL4*	0.290
7	*GAPDH*	0.352	*GAPDH*	0.388	*ALB*	0.300
8	*B2M*	0.400	*B2M*	0.442	*B2M*	0.370
9	*CA2*	0.440	*CA2*	0.489	*CA2*	0.500
10	*ACTB*	0.482	*ACTB*	0.551	*ACTB*	0.500

### Effect of time-points and nicarbazin treatment on stability of reference gene expression (Experiment 2)

The overall expression pattern (Cq values) of all the ten reference genes is depicted in Fig 3D. Calculating the expression stability of the reference genes for both the groups combined (control and nicarbazin treated), geNorm ranked *HMBS* and *HPRT1* as the two most stable genes (Table 6). The pairwise variations (geNorm V) for the control, nicarbazin treated and both the groups combined are shown in Fig 3E. The geNorm V values of the pairwise variation of the ten reference genes for the control, nicarbazin and both the groups combined were <0.15 (a default cut-off value). In order to gain insight into any difference between the expression data of the time-points experiment (Experiment 1) and the combination of time-points and nicarbazin treatment experiment (Experiment 2), the expression data of time-points and the control hens from time-points and nicarbazin treatment were analysed together with the exclusion of post-oviposition time 2 hours. The gene ranking was reshuffled and *HMBS* was ranked as the least stable gene (Fig 3F). NormFinder ranked *HMBS* and *RPL4*, while the BestKeeper showed *HPRT1* and *HMBS* as the two most stable genes (Table 6). Categorising all the ten reference genes into most stable, middle order and least stable, the reshuffling in gene ranking was different but relatively consistent for the first four genes across all the three statistical tools. It seems that the genes falling in mid order were more variable in stability when analysed for comparison by the three statistical tools. For the overall ranking obtained by the three algorithms, the two most stable reference genes for the total dataset were *HMBS* and *HPRT1*, while the two least stable genes were *B2M* and *CA2* (Table 6).

**Table 6:** Overall stability values of reference genes affected by time-points and nicarbazin treatment. Means of relative expression levels in respective groups at three different time-points (post-oviposition times 5, 15, 23.5 hrs) and with nicarbazin treatments (yes, no) were used to calculate the expression stability of genes in responses to the time points and nicarbazin treatment across the three statistical software.

Rank	geNorm		NormFinder		BestKeeper	
	Gene	M value	Gene	SD	Gene	SD
1	*HMBS*	0.236	*HMBS*	0.182	*HPRT1*	0.210
2	*HPRT1*	0.243	*RPL4*	0.191	*HMBS*	0.220
3	*GAPDH*	0.247	*HPRT1*	0.266	*RPL4*	0.230
4	*RPL4*	0.300	*18S rRNA*	0.291	*GAPDH*	0.270
5	*18S rRNA*	0.331	*GAPDH*	0.296	*ALB*	0.320
6	*ALB*	0.369	*ALB*	0.409	*18S rRNA*	0.330
7	*ACTB*	0.418	*ACTB*	0.446	*ACTB*	0.380
8	*CST3*	0.454	*CST3*	0.475	*CST3*	0.450
9	*CA2*	0.488	*CA2*	0.491	*CA2*	0.480
10	*B2M*	0.601	*B2M*	0.991	*B2M*	0.860

When the data were normalized in geNorm for each group separately, the control group showed that *HMBS* and *HPRT1* were the most stable reference genes (Table 7). In the same group, both NormFinder and BestKeeper ranked *RPL4* and *HMBS* as the two most stable reference genes. However, the overall ranking of the genes falling in middle order was reshuffled following the analyses by all the three statistical software. In the control group, the two least stable genes across all the three applets were *B2M* and *CA2*. The only genes that showed higher M value than the cut-off value in geNorm were *CA2* and *B2M* (Table 7).

**Table 7:** Stability values of reference genes affected by time-points. Means of relative expression levels of the genes in the control group at three different time-points (post-oviposition times 5, 15, 23.5 hrs) were used to calculate the expression stability of genes across the three statistical software.

Rank	geNorm		NormFinder		BestKeeper	
	Gene	M value	Gene	SD	Gene	SD
1	*HMBS*	0.207	*RPL4*	0.138	*RPL4*	0.190
2	*HPRT1*	0.207	*HMBS*	0.172	*HMBS*	0.220
3	*GAPDH*	0.214	*HPRT1*	0.225	*HPRT1*	0.240
4	*RPL4*	0.274	*18S rRNA*	0.300	*GAPDH*	0.260
5	*18S rRNA*	0.305	*GAPDH*	0.309	*18S rRNA*	0.290
6	*ACTB*	0.373	*CST3*	0.455	*ALB*	0.340
7	*CST3*	0.424	*ACTB*	0.459	*ACTB*	0.400
8	*ALB*	0.461	*ALB*	0.499	*CST3*	0.450
9	*CA2*	0.503	*CA2*	0.544	*CA2*	0.480
10	*B2M*	0.609	*B2M*	0.963	*B2M*	0.710

The reference genes ranking in the nicarbazin treatment group was slightly different from the control group. The geNorm ranked *HMBS* and *GAPDH* as the two most stable genes followed by *HPRT1* (Table 8). NormFinder showed *HMBS* and *ALB*, while the BestKeeper showed *HPRT1* and *HMBS* as the two most stable reference genes (Table 8). The two least stable genes across all the three applets were *B2M* and *CST3* (Table 8). The stability values of *B2M* were slightly higher than the cut-off value (geNorm M < 0.5; SD < 1.0) when the data were analysed both in the geNorm and NormFinder.

**Table 8:** Stability values of reference genes affected by nicarbazin treatment (nicarbazin group only). Means of relative expression levels of the genes in the nicarbazin group at three different time-points (post-oviposition times 5, 15, 23.5 hrs) were used to calculate the expression stability of genes across the three statistical software.

Rank	geNorm		NormFinder		BestKeeper	
	Gene	M value	Gene	SD	Gene	SD
1	*HMBS*	0.262	*HMBS*	0.199	*HPRT1*	0.190
2	*GAPDH*	0.273	*ALB*	0.226	*HMBS*	0.230
3	*HPRT1*	0.278	*RPL4*	0.231	*GAPDH*	0.260
4	*ALB*	0.329	*HPRT1*	0.284	*RPL4*	0.280
5	*RPL4*	0.340	*GAPDH*	0.289	*ALB*	0.280
6	*18S rRNA*	0.360	*18S rRNA*	0.297	*ACTB*	0.300
7	*ACTB*	0.403	*CA2*	0.427	*18S rRNA*	0.340
8	*CA2*	0.435	*ACTB*	0.435	*CA2*	0.460
9	*CST3*	0.463	*CST3*	0.513	*CST3*	0.490
10	*B2M*	0.583	*B2M*	1.008	*B2M*	0.860

**Fig 4:**
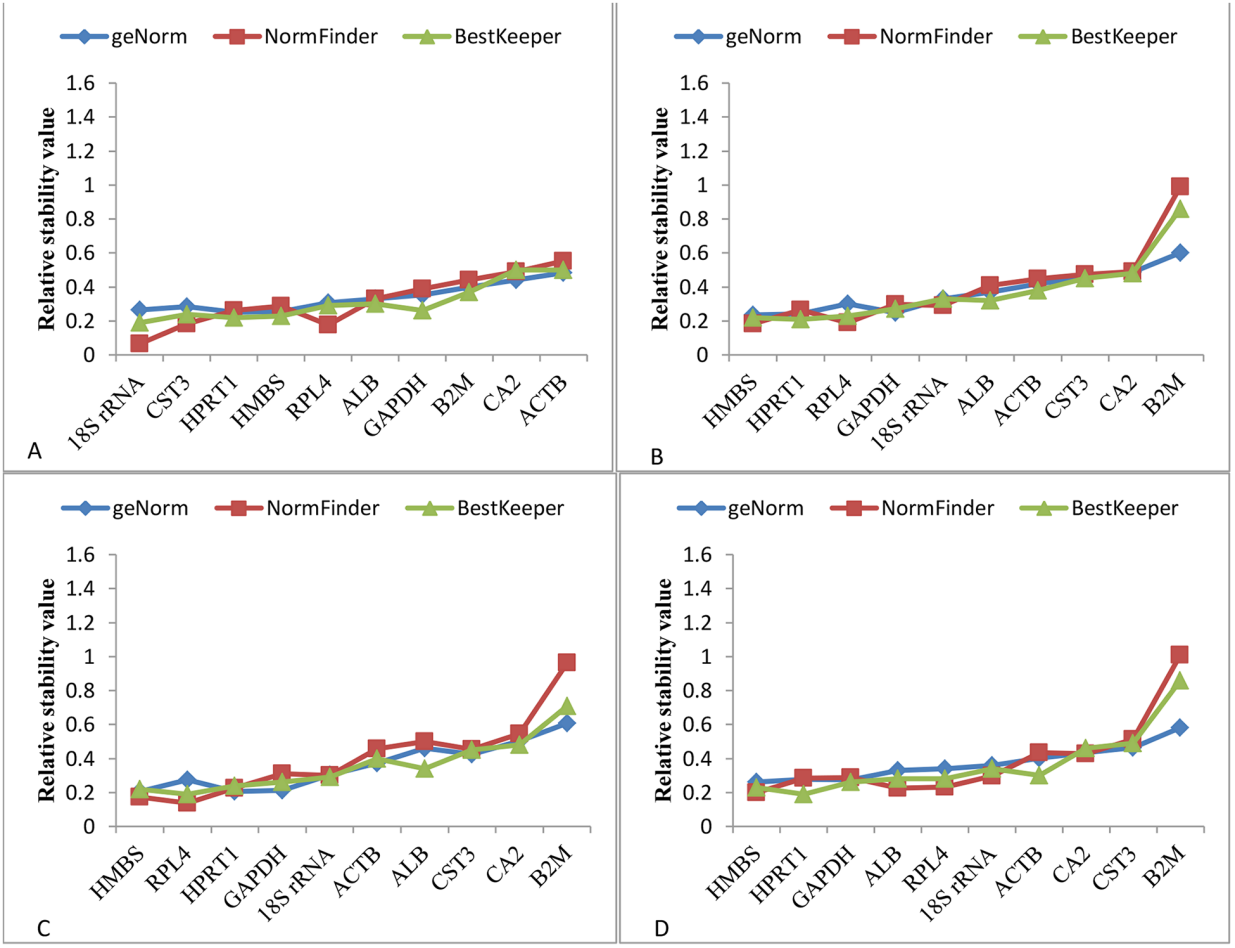
Comparison of the relative expression stability of 10 candidate reference genes analysed by geNorm, NormFinder and BestKeeper. The geNorm M and SD values of 10 reference genes were graphed to assess the ranking pattern of the genes assigned by three different software. A). Four different time-points (2, 5, 15, 23.5 hr) post-oviposition in the birds at age of 36–37 weeks. B). Groups treated with or without nicarbazin at three different time-points (5, 15, 23.5 hr) post-oviposition of birds at 42–45 weeks. C). Three different time-points (5, 15, 23.5 hr) post-oviposition times of the birds without nicarbazin treatment at age of 42–45 weeks. D). Three different time-points (5, 15, 23.5 hr) post-oviposition of the birds with nicarbazin treatment at age of 42–45 weeks.

In order to determine the consistency of the stabilities of the reference genes analysed by geNorm, NormFinder and BestKeeper, the relative expressions of the ten genes were compared as shown in Fig 4. The results showed that the expression stability of the genes was consistent from the analyses performed by the three programs except *B2M* when the birds were treated with nicarbazin at three time-points of egg shell formation (birds age 42–45 weeks).

### Expression of candidate target genes using most stable and least stable reference genes

**Fig 5:**
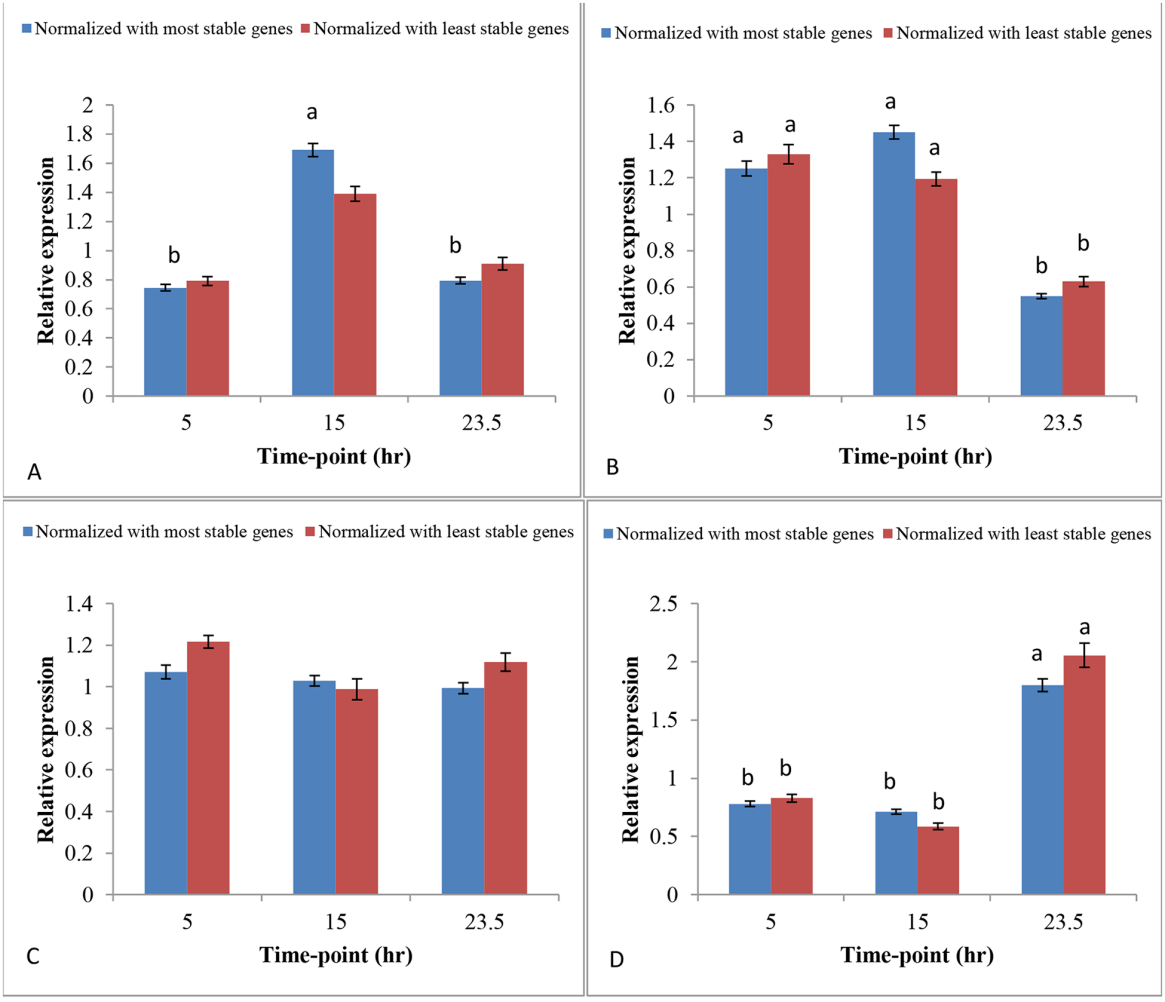
Validation of the expression level of four candidate target genes affected by three different time-points of eggshell formation. The candidate target genes were normalized with the two most stable (*HMBS* and *HPRT1*) and the two least stable reference genes (*B2M* and *CA2*). A). *SLC25A38* normalized with the two most stable genes (P = 4.8E-10); *SLC25A38* normalized with the two least stable genes (P = 0.0847). B) *ALAS1* normalized with the two most stable genes (P = 3.2E-12); *ALAS1* normalized with the two least stable genes (P = 0.0111). C). *CPOX* normalized with the two most stable genes (P = 0.0700); *CPOX* normalized with the two least stable genes (P = 0.6134). D). *FECH* normalized with the two most stable genes (P = 4.6E-16); *FECH* normalized with the two least stable genes (P = 9.9E-05). For the same gene in the same treatment, ^a,b^ across the bars indicate significant differences. B). For the four candidate target genes, normalized relative quantities were calculated in qbase+ based on (2^^-ΔΔCq^) [40] using gene specific amplification efficiencies [41], to show the relative expression of Cq levels in folds to the mean Cq of all samples of the genes.

The level of significance (p value) changed for all four candidate target genes when the relative expression data were normalized with the two most stable (*HMBS*, *HPRT1*) and the two least stable reference genes (*B2M*, *CA2*) (Fig 5). The p value increased when the data were normalized with the two least stable reference genes. The relative expression level of *SLC25A38* was significantly different (p value 4.8E-10) among different time-points when the data were normalized with the two most stable reference genes (*HMBS* and *HPRT1*) (Fig 5A). However, for the same candidate target gene, the level of significance decreased (p value = 0.0847) among different time-points when the data were normalized with the two least stable reference genes, *B2M* and *CA2* (Fig 5A). The expression levels of *ALAS1*, *CPOX* and *FECH* were changed in terms of the p values when the data were normalized with the two most stable and the two least stable reference genes. The p value of *ALAS1* changed from 3.2E-12 when two most stable reference genes were used, to 0.0111 when the two least stable reference genes were used (Fig 5B). The p value of *CPOX* changed from 0.0700 to 0.6134 when the data were normalized with the two least stable reference genes (Fig 5C). The p value of *FECH* changed from 4.6E-16 to 9.9E-05 when the data were normalized with the two least stable reference genes (Fig 5D).

## Discussion

We investigated the stability of ten reference genes in the shell gland region of the oviduct in relation to different stages of eggshell formation and nicarbazin treatment. The data analysed by the three different statistical software indicated that the overall stability of the reference genes was affected by different time-points (post-oviposition time) and nicarbazin treatment, and differences in the ranking of reference genes analysed using three statistical software were observed. The current study provides information on the expression stability of these candidate reference genes and most stably expressed reference genes are suggested for the normalization of gene expression data in the chicken shell gland.

The higher stability of *HPRT1* and *HMBS* across all the three software indicated that these two genes can be used as reference genes for the normalization of expression data in the shell gland of the brown-egg laying hen. In fact, most of the ten reference genes tested in the current study were in the acceptable range as reference genes with geNorm M value <0.5 and SD <1.0. *B2M* was the only exception that showed slightly higher geNorm M value in the time-points and nicarbazin treatment study. Taking time-points separately, or together with the nicarbazin treatment, the pairwise variation (geNorm V) showed that the variation between the first two most stable genes was under the cut-off value (<0.15). In qbase+, geNorm V indicates level of variation in the average values of reference gene stability with the sequential inclusion of the next stable reference gene to the equation Vn/n+1 (for calculation of the normalization factor). The analysis starts with the two most stably expressed genes being compared to the pair including the third (V2/3), and the process continues until the least stable gene is added (for example, V9/10). Generally, if a geNorm V (V2/3) of 0.300 is achieved using two most stable genes and a geNorm V (V3/4) of 0.14 is achieved with three most stable reference genes, then the average of the most stable three genes would be the optimal normalization factor for further data analysis. In the current study, the first two most stable reference genes were under the cut-off value (<0.15) of geNorm V and thus adding the third most stable reference gene for expression data normalization was not necessary. However, as indicated by the geNorm V, all of the genes (V2/3 to V9/10) showed pairwise variation < 0.15 and thus all could be used for accurate data normalization. Based on the geNorm V results, this demonstrates that all the genes analysed had relatively high stability in the shell gland tissue in response to nicarbazin treatment of chickens as well as to the cyclic changes in shell gland tissue during the egg lying cycle. Based on geNorm M results, *B2M* showed low expression stability in response to the stages of egg formation in the nicarbazin treatment experiment and therefore should be ruled out from being used in the normalisation of expression data while different stages of egg formation are involved in the study.

Furthermore, the difference in stability values of *B2M* during the egg formation stages may also be dependent on the age of the birds. It appears that it is more stable in response to the time-points when birds are younger (36–37 week vs 42–45 week of age). Nevertheless, consensus from the analyses performed by these three programs was that *HMBS* and *HPRT1* were the two most stable housekeeping genes and thus were chosen as reference genes in the current study and recommended for similar studies in the shell gland of laying hens.

To validate whether most stable reference genes identified in the study would result in more accurate assessment of target gene expression, the data obtained in time-points and nicarbazin treatment experiment for four candidate target genes were normalized with the two most stable (*HPRT1*, *HMBS*) and the two least stable reference genes (*B2M*, *CA2*). Results showed that normalizing candidate target gene expression data with the two least reference stable genes is not as accurate when compared with the data normalized with the two most stable reference genes. Therefore, the most stable genes can produce more robust and accurate results for gene expression data as recommended by the optimisation outcomes achieved in the current study.

To the best of our knowledge, no validated reference genes have been used for the normalization of expression data in the shell gland of avian species under various treatments. Thus, this is the first study to establish a set of stably expressed reference genes in the shell gland and can be used in chickens and possibly in other avian species. In different species, different reference genes under different treatments have been validated in other tissues of the reproductive system. For example, in geese, *HPRT1* and *GAPDH* were ranked as the two most stable reference genes in the ovary [42]. In bovine, the two most stable reference genes in the uterus were *YWHAZ* and *GAPDH* in relation to developmental stages of an embryo [43]. Similarly, under various toxicological treatments, in the ovary of mouse, the two most stable reference genes were *RPL13a* and *GAPDH* [44]. Based on limited studies already being performed, it appears *HPRT1* may be more stable in the reproductive system of avian species while *GAPDH* is more stable in mammalian species [40]. However, further studies are required to accumulate the information required to reach a more generalised conclusion.

The present study has demonstrated that the rankings of the expression stability of the 10 candidate reference genes had similar trends but discrepancies were observed among three different statistical programs, geNorm, NormFinder and BestKeeper. Similar discrepancies have been observed elsewhere with different species and treatments [42, 45, 46]. So far, there is no consensus as to which software is more powerful in the ranking of expression stability of candidate reference genes and researchers have given the same weight to all three programs. We have shown in this study a comparison in the consistency of the ranking of candidate reference genes among all the three software used in both experiments, indicating that all of them gave similar results and can be used for the analysis of expression data. As has been stated previously, the stability of all the chosen reference genes was in the acceptable range for reference genes. Therefore, the stability levels of these genes are essentially very close. With different algorithms in different programs, slight change of their stability orders can be expected by the analyses using these programs.

It is worth noting; however, that these three programs do not have an option for analysing the reference gene expression data generated from a factorial design, but can only perform analysis based on individual group as independent treatment. To the best of our knowledge, the optimisation of reference genes has not been performed in such a factorial design so far. Therefore, it is questionable whether the programs possess the capacity to generate a reliable ranking for an experiment designed in a factorial fashion. The gene expression stability analysis of reference genes has been reported in experiments exploring the roles of multiple factors; for example, geographical locations and ventilation in new born lambs [47], multiple stress conditions and different developmental tissues in pear millet [48] and different developmental stages and hormonal stimuli on leaves of tea [49]. However, multiple factors have not been considered as independent effects in the analysis of stability of reference gene expression or for their interactions. We suggest these programs should add such a capacity or new programs should be available for the analyses of data produced from factorial design experiments. This would permit the role of treatments in the expression stability of the reference genes to be more robustly investigated and their interactions explored. Such investigations are warranted to provide a more powerful statistical analysis protocol.

## Conclusion

In summary, we have performed optimisation of reference genes in the samples collected at different time-points of egg/eggshell formation and with nicarbazin treatment in laying hens. All of the reference genes except *B2M* were stably expressed according to the cut-off values of the programs, and two most stably expressed genes, *HMBS* and *HPRT1* are recommended for the normalization of gene expression data in the shell gland of chickens while different shell formation stages and nicarbazin treatment are involved in the experiment. It is anticipated that these reference genes may be used for the study of reproductive system in other avian species.
